# Sugarcane bagasse ash as fertilizer for soybeans: Effects of added residues on ash composition, mineralogy, phosphorus extractability and plant availability

**DOI:** 10.3389/fpls.2022.1041924

**Published:** 2022-12-08

**Authors:** Vitalij Dombinov, Hannes Herzel, Martin Meiller, Felix Müller, Sabine Willbold, Joachim W. Zang, Warde A. da Fonseca-Zang, Christian Adam, Holger Klose, Hendrik Poorter, Nicolai D. Jablonowski, Silvia D. Schrey

**Affiliations:** ^1^ Institute of Bio- and Geosciences, IBG-2: Plant Sciences, Forschungszentrum Jülich GmbH, Jülich, Germany; ^2^ Thermochemical Residues Treatment and Resource Recovery, Bundesanstalt für Materialforschung und -prüfung (BAM), Berlin, Germany; ^3^ Renewable Energy, Fraunhofer Institute for Environmental, Safety, and Energy Technology UMSICHT, Sulzbach-Rosenberg, Germany; ^4^ Thermal Process Technology, TU Clausthal (CUTEC), Clausthal-Zellerfeld, Germany; ^5^ Central Institute for Engineering, Electronics and Analytics, Analytics (ZEA-3), Forschungszentrum Jülich GmbH, Jülich, Germany; ^6^ Instituto Federal de Educação, Ciência e Tecnologia de Goiás (IFG), Goiânia, Brazil; ^7^ RWTH Aachen University, Aachen, Germany; ^8^ Department of Natural Sciences, Macquarie University, Sydney, Australia

**Keywords:** combustion and gasification, ^31^P-NMR spectroscopy, X-ray diffraction (XRD), phosphate extractability and availability, greenhouse experiments

## Abstract

Sugarcane bagasse is commonly combusted to generate energy. Unfortunately, recycling strategies rarely consider the resulting ash as a potential fertilizer. To evaluate this recycling strategy for a sustainable circular economy, we characterized bagasse ash as a fertilizer and measured the effects of co-gasification and co-combustion of bagasse with either chicken manure or sewage sludge: on the phosphorus (P) mass fraction, P-extractability, and mineral P phases. Furthermore, we investigated the ashes as fertilizer for soybeans under greenhouse conditions. All methods in combination are reliable indicators helping to assess and predict P availability from ashes to soybeans. The fertilizer efficiency of pure bagasse ash increased with the ash amount supplied to the substrate. Nevertheless, it was not as effective as fertilization with triple-superphosphate and K_2_SO_4_, which we attributed to lower P availability. Co-gasification and co-combustion increased the P mass fraction in all bagasse-based ashes, but its extractability and availability to soybeans increased only when co-processed with chicken manure, because it enabled the formation of readily available Ca-alkali phosphates. Therefore, we recommend co-combusting biomass with alkali-rich residues to increase the availability of P from the ash to plants.

## Introduction

In tropical and subtropical countries, the combustion of sugarcane bagasse and leaves is used to generate heat and electricity (Raza et al., 2021). This combustion can be considered sustainable (Ondrasek et al., 2021), as it releases only carbon that has been sequestered from the atmosphere (Mendiara et al., 2017). In Brazil, about 6% of electricity is generated from the combustion of sugarcane leaves and bagasse (Watanabe et al., 2020), resulting in up to 10 million tons of ash annually (Herzel et al., 2020). However, sustainability also depends on appropriately recycling valuable elements found in the ash, such as phosphorus (P) and potassium (K). Although bagasse ash has been recognized as a potential fertilizer because it contains P and K (Pita et al., 2012; Gonfa et al., 2018), it is often disposed of as waste in landfills (Xu et al., 2018; Silva et al., 2019). This is due to both a lack of reliable information on nutrient availability compared to conventional fertilizers, and because the ash is partly produced in the rainy season, which makes transport to the field difficult. Biomass combustion and use of the resulting ash in agriculture is a promising strategy for sustainable energy production, disposing of ash at low-cost while recycling valuable nutrients (Patterson et al., 2004). Ultimately, such practices will help to achieve global goals for climate change mitigation by making efficient use of resources (EU, 2016; Li et al., 2016; Müller-Stöver et al., 2018; EU, 2019; Chojnacka et al., 2020).

While K from biomass ash is comparable to that found in mineral fertilizers (Li et al., 2017; Tran et al., 2018), the availability of P from ashes to plants depends, among other factors, on the origin and composition of the biomass. This can be modified by joint processing with other (nutrient-rich) residues, and by varying thermal processing conditions (Demeyer et al., 2001; Schiemenz and Eichler-Löbermann, 2010; Müller-Stöver et al., 2018). Following thermal processing, organic P oxidizes and reacts with other metals, including alkaline earth metals and alkali metals such as aluminum (Al) and iron (Fe), calcium (Ca), and sodium (Na), forming a mixture of oxides and phosphates (Tan and Lagerkvist, 2011). The solubility of phosphates from ash and their availability to plants depends on mineral P types formed during the thermal processing of biomass (Brod et al., 2015; Kratz et al., 2019; Luyckx et al., 2021). To our knowledge, there is currently only limited information available on the P mineralogy of bagasse ash, and thus the fertilization effects of these ashes are not well understood.

The objectives of the present study were to produce ashes by combusting and gasifying sugarcane bagasse alone and in combination either with chicken manure or sewage sludge. The goal of these variations was to i) change the elementary composition of bagasse ash and ii) use laboratory and greenhouse analyses to understand and predict plant P availability as well as subsequent fertilization effects. Chicken manure and sewage sludge were used because they both have high P mass fractions, while otherwise differing in Ca, Na, K, Fe, and Al content. The co-processing also aimed to show the benefits of using bagasse ash as a fertilizer. We chose soybean as a model plant because it is one of the most widely cultivated crops in Brazil (FAOSTAT, 2022) and is often cultivated in rotation with sugarcane (Bordonal et al., 2018; Withers et al., 2018). In this study, we conducted laboratory and greenhouse experiments to understand and predict the plant P availability from different bagasse-based ashes and their fertilization effects on soybeans.

## Material and methods

### Ash preparations

Bagasse ashes from the sugarcane factory ′Usina Nova Gália Ltda.′ (Paraúna, Goiás state, Brazil), hereafter named cB ash, as well as five bagasse-based ashes resulting from small-scale combustion and gasification, were used in this study. On a small-scale, bagasse pellets were gasified alone (gB) or as a blend with chicken manure pellets (gBM), and co-combusted either with chicken manure pellets (cBM) or sewage sludge (cBS; **Supplementary Material S1, S2**). The ratios of bagasse pellets to chicken manure pellets and sewage sludge were 80 to 20 (w/w). All sugarcane bagasse-based ashes were dried to constant weights at 60°C (TR 1050, Nabertherm GmbH, Lilienthal, Germany). The ashes, as well as triple superphosphate (TSP; Triferto Fertilizers, Doetinchem, Netherlands), were milled (≤ 250 μm, Retsch ZM 1, Retsch GmbH, Haan, Germany) before analyses and use in greenhouse pot experiments.

### Ash characterizations

#### Elementary compositions

All bagasse-based ashes and TSP were analyzed for their elemental composition. Carbon (C) and nitrogen (N) were measured in a Vario El Cube, CHNS elemental analyzer (CHNS mode, Elementar Analyzensysteme GmbH, Langenselbold, Germany) by combusting 0.1 g of each fertilizer. Another 0.1 g of fertilizer was mixed with 0.25 g lithium borate and digested at 1000°C in a muffle furnace for 30 min. The molten phase was diluted in 30 ml of 5% hydrochloric acid (HCl) and filled up to 50 ml with ultra-pure water (Milli-Q Reference, Merck, Darmstadt, Germany). Subsamples for P, K, Ca, Na, Fe, Al, magnesium (Mg) and silicon (Si) determination were then diluted with ultra-pure water at a ratio of 1:20 (*v*/*v*) and measured in inductively coupled plasma atomic emission spectroscopy (iCAP 6500, Fisher Scientific, Schwerte, Germany).

#### Sequential phosphorus extraction

The sequential P extractions from cB, gB, gBM, cBM and cBS ashes followed the protocol of Hieltjes and Lijklema (1980), modified by Qian et al. (2009). In brief, 0.5 g of each ash were sequentially extracted with 20 mL of distilled deionized water (ddH_2_O), 1 M ammonium chloride (NH_4_Cl) at pH 7, 0.1 M sodium hydroxide (NaOH), and twice with 0.5 M hydrochloric acid (HCl) solutions. Samples were shaken (Turbula T2c, Willy A. Bachofen AG, Muttenz, Switzerland) for 2 hours (h), 2 h, 17 h, and 2x 24 h, respectively, and centrifuged at 15000 *g* (Hermle Z326K, HERMLE Labortechnik GmbH, Wehingen, Germany) for 5 min. before collecting supernatants. Phosphorus in supernatants was measured using inductively coupled plasma atomic emission spectroscopy (Thermo iCAP 7400, Dreieich, Germany) according to Brod et al. (2015).

#### Phosphorus and potassium extraction in citric acid

Phosphorus and K were extracted with 2% citric acid from all bagasse-based ashes and TSP following the protocol of Herzel et al. (2020). Briefly, 1.0 g ± 0.1 g of ashes or TSP were mixed with 100 mL of 2% citric acid solution (1:100 *w/v*), then shaken for 30 min. in an overhead shaker (35 rpm, RA20, Gerhardt, Königswinter, Germany) and filtered *via* a folded filter (type 2015, particle retention 5-8 µm, Labsolute, Renningen, Germany). Phosphorus and K concentrations in the filtrates were analyzed by inductively coupled plasma atomic emission spectroscopy (Thermo iCAP 7400, Dreieich, Germany).

#### Powder X-ray diffraction analysis

The powder X-ray diffraction (XRD) measurements were based on the protocol described by Herzel et al. (2020). Measurements focused on P phases in cB, gB, gBM, cBM and cBS ashes and were combined with P extractions in 2% citric acid, 2% formic acid, 0.1 M NaOH and 0.5 M HCl to verify the disappearance of reflexes due to pH changes. Ash measurements were performed in Bragg-Brentano geometry over a 2θ range from 5° to 80° with a step size of 0.02° employing a D8 Advance Bruker AXS (Bruker, Billerica, USA) before and after P extraction in 0.1 M NaOH and 0.5 M HCl solutions, respectively. Diffraction patterns were collected using Cu Kα1 and Cu Kα2 (λ1 = 1.54056 Å, λ2 = 1.54443 Å) radiation and recorded with a Lynxeye detector. Qualitative identification of the crystalline phases was performed using the MATCH! Software (version 3.6) in combination with the ICDD PDF2 database.

### Plant trials under greenhouse conditions

Two independent greenhouse pot experiments were conducted at the Institute of Bio- and Geosciences, IBG-2: Plant Sciences, Forschungszentrum Jülich, Germany (50°54’36’’N, 6°24’49’’E), to evaluate the fertilizing effect (Exp. 1) and plant P availability (Exp. 2) from bagasse-based ashes to soybeans.

Soybeans (*Glycine max* (L.) Merr., RGT Shouna) were cultivated as described in Herzel et al. (2020) to provide homogeneous plant material at the start of the experiments. Soybeans were germinated on moist filter paper in the dark, and after 3 days (d) seedlings with comparable radicle length were transplanted into the substrate, i.e., sand and a greenhouse substrate low in nutrients (*Null-Erde*, Balster Einheitserdewerk, Fröndenberg, Germany) in a volume ratio of 1:1 (**Supplementary Material S3**) until the unifoliate leaves were fully expanded. At this stage, the roots were washed and plantlets with comparable morphologies were identified. Five plantlets were harvested to analyze the initial K and P contents (referred to as the first harvest in Eq. 2 and 3). Roots of remaining plantlets were inoculated with N_2_-fixing *Bradyrhizobium japonicum* (NPPL HI Stick, BASF SE, 180 Ludwigshafen, Germany). Plantlets were then transplanted into pots containing 1150 gram (g), i.e. 1.25 liter (L), of substrate treated with the different fertilizers. The selection of the potting medium was based on additional analyses (**Supplementary Material S3**). The substrate chosen as the potting medium was selected because it contained only a small mass fraction of P, and its physical and chemical properties were not significantly affected by high doses of bagasse ash, unlike either Brazilian soil from the field or quartz sand. This enabled analyses of ash as fertilizer and of P availability from ashes to soybeans under highly controlled conditions.

Soybean plants received 16 h d^-1^ light from natural and artificial light sources (minimum 400 μmol s^-1^ m^-^², SON-T AGRO 400, Philips) regulated by an automated light system. The pots were watered to 70% of their water holding capacity weekly. The substrate was covered with white plastic granulate to reduce water evaporation. Pots were randomized each week to minimize edge effects.

#### Experiment 1: Soybean response to increasing doses of cB ash

A dose-response experiment using cB ash (**Table 1**) as fertilizer for soybeans was conducted from August to September. The aim of this experiment was to analyze the efficiency of pure bagasse ash as fertilizer for soybeans under greenhouse conditions. Six doses of cB ash, ranging from 0 g to 31.6 g cB ash kilogram^-1^ (kg^-1^) substrate, were homogeneously incorporated into the substrate and delivered 0 mg to 120 mg P and 0 mg to 385 mg K kg^-1^ of substrate. Positive control treatment received readily available P and K at optimal doses for soybeans, i.e., 30 mg P kg^-1^ from TSP and 225 mg K kg^-1^ from potassium sulphate (K_2_SO_4_), determined in pre-experiments (data not shown). According to the recommendation for soybean cultivation under field conditions, soybean plantlets were inoculated with the symbiotic microbe *Bradyrhizobium japonicum* (2.3) and no mineral N fertilizer was added (Mendes et al., 2003).

**Table 1:** Chemical compositions of cB, gB, gBM, cBM and cBS ashes and triple superphosphate (TSP).

Elements	Unit	cB ash	gB ash	gBM ash	cBM ash	cBS ash	TSP
		B	B	B/M (80:20)	B/M (80:20)	B/S (80:20)	
		Comb.	Gas.	Gas.	Comb.	Comb.	
**C**	wt%	5.05	53.7	40.5	1.07	0.21	
**N**	wt%	0.1	0.05	0.08	0.03	0.02	
**P**	wt%	0.38	0.41	1.66	2.05	3.02	19.48
**K**	wt%	1.21	1.04	2.41	3.15	0.64	0.04
**Ca**	wt%	1.26	0.99	5.15	9.12	3.99	13.74
**Mg**	wt%	0.46	0.57	1.14	1.01	0.67	0.23
**Na**	wt%	0.1	0.04	0.19	0.24	0.11	
**Fe**	wt%	5.38	3.32	2.46	1.44	3.86	0.28
**Al**	wt%	3.86	2.98	2.72	1.14	4.18	0.23
**Si**	wt%	14.62	0.56	0.72	0.04	0.01	
**pH_1:2.5_**		7.1	9.9	11.5	12.8	7.7	2.6
**P_CA_**	mg P g^-1^	1.86	3.97	15.6	17.34	9.02	183.09
**K_CA_**	mg K g^-1^	6.05	3.64	14.46	16.7	-	-

The pH values were measured in 0.01 M CaCl_2_ solution (1:2.5 w/v). P_CA_ and K_CA_ indicate the mass fractions of P and K from ash, TSP and K_2_SO_4_ soluble in 2% citric acid. B, bagasse; M, chicken manure; S, sewage sludge; Comb., combustion; Gas., gasification.

Each fertilizer treatment contained five biological replicates, which were harvested after 44 d of growth. During plant growth the average relative air humidity was 52/66% (day/night) and the temperature was 25/20°C (day/night). The plants received an average of 9.8 mol photons m^-2^ d^-1^ light irradiance.

#### Experiment 2: Effect of thermal co-processing bagasse pellets with nutrient rich residues on P-availability to soybeans

Over the period from April to June, we evaluated the P availability from gB, gBM, cBM and cBS ashes (**Table 1**) to soybeans. The soybeans were fertilized with 54 mg P kg^-1^ of substrate. Phosphorus dose was based on the dose response experiment discussed in Effect of industrially produced bagasse ash as fertilizer. Absolute amounts of applied bagasse-based ashes were 13.2 g of gB, 3.2 g of gBM, 2.2 g cBM, and 1.9 g cBS ashes kg^-1^ substrate. For all treatments, the concentration of K was adjusted with K_2_SO_4_ to 244 mg kg^-1^ and with ammonium nitrate to 23 mg of N kg^-1^ of substrate to exclude K and N limitation when comparing plant P availability from gB, gBM, cBM and cBS ashes.

Each fertilizer treatment contained five biological replicates, which were harvested after 42 d of growth. Average relative air humidity was 47/62% (day/night) and the temperature was 25/19°C (day/night). The plants received an average of 11.2 mol photons m^-2^ d^-1^ light irradiance.

#### Non-invasive shoot growth measurements

In Experiment 1, the shoot areas of soybean plants were regularly imaged using the plant phenotyping “ScreenHouse” platform available at IBG-2 Plant Sciences, Forschungszentrum Jülich GmbH, Germany, as described in Nakhforoosh et al. (2016) and Herzel et al. (2020) to depict the shoot growth dynamics. In brief, the shoots were automatically imaged at a 45° angle from four sides of the pot, i.e., 0°, 90°, 180°, and 270° (Point Gray Grasshopper2, 5MP color camera, by FLIR Integrated Imaging Solutions Inc., Richmond, British Columbia, Canada). The sum of the four images represented the projected shoot area of the plant. Shoot areas were imaged weekly for 26 d, starting on day 6 after transplanting the plantlets into the fertilized substrate. Imaged shoot area was significantly correlated with leaf area of soybeans after plant harvest (R = 0.96, **Supplementary Material S4**), allowing an estimation of projected leaf area over time. The factor used to convert the projected shoot areas from px to projected leaf areas in cm^2^ was 1792 and was based on a calibration curve (**Supplementary Material S4**).

#### Destructive analyses

In experiment 1, roots and shoots were harvested separately and numbers of root nodules were counted to evaluate the evenness of root nodulation between treatments. In both experiments 1 and 2, roots and shoots were separately dried at 65°C to constant weight (TR 1050, Nabertherm GmbH, Lilienthal, Germany). The dry masses (DM) were determined (PG503-S, Mettler Toledo GmbH, Gießen, Germany) and the root mass fractions (RMF) were calculated, defined as dry mass of nodulated roots (DM_Roots_) relative to the total dry mass (DM_Total_) of the plant from shoot and root organs (Eq. 1).


(Eq. 1) $$RMF\;(g\;g^{-1})=\frac{DM_{Root}\;\left(g\right)}{\;DM_{Total}\left(g\right)}$$

Before washing the roots, 250 g of homogenized substrate were sampled, dried at 30°C to constant weight and used for pH measurements (**Supplementary Material S5**).

#### Chemical biomass analyses

Shoot and root material was ground (MM 400, Retsch GmbH, Haan, Germany) separately in experiment 1, and collectively in experiment 2. Phosphorus and K were analyzed as follows: 50 g of dry biomass were digested in a microwave (Mars 5, Prg. Pflanzen160 X-Press, Kamp-Lintfort, Germany) with 2 mL of HNO_3_ and 1 mL of H_2_O_2_ for 35 min. including heating and residence time in triplicate. The samples were then diluted with ultra-pure water (Milli-Q Reference, Merck, Darmstadt, Germany) to a total volume of 14 mL. Before measurement by inductively coupled plasma atomic emission spectroscopy (iCAP 6500, Fisher Scientific, Schwerte, Germany), subsamples were diluted to a ratio of 1:20. The subsamples were measured for P in both experiments and additionally for K in experiment 1, which focused on the fertilization effect of cB ash as a PK fertilizer rather than only on plant P availability from the ashes as in experiment 2. Phosphorus and K uptakes were calculated as described by Herzel et al. (2020), i.e., as the differences in total P and K in total dry matter of soybeans after 44 d (see Experiment 1: Soybean response to increasing doses of cB ash) and 42 d (see Experiment 2: Effect of thermal co-processing bagasse pellets with nutrient rich residues on P-availability to soybeans) of growth in fertilized substrates (referred to as final harvest) and before fertilization beginning (see Plant trials under greenhouse conditions), i.e., first harvest (Eq. 2 and Eq. 3).


(Eq. 2) $$\Delta P_{Uptake}\;\left(mmol\right)=P_{final\;harvest}\;\left(mmol\right)-\;P_{first\;harvest}\;\left(mmol\right)$$

(Eq. 3) $$\Delta K_{Uptake}\;\left(mmol\right)=K_{final\;harvest}\;\left(mmol\right)-\;K_{first\;harvest}\;\left(mmol\right)$$

To evaluate the potential substitution of commercial P and K from TSP and K_2_SO_4_ by cB ash, the relative agronomic effectiveness (RAE) of P and K from cB ash was calculated according to Bogdan et al. (2021). RAE is based on the uptakes of P and K from cB ash (P_cB_), TSP (P_TSP_), K_2_SO_4_ (K_K2SO4_) and unfertilized substrate (P_0_, K_0_) following the Eq. 4 and Eq. 5.


(Eq. 4) $$RAE\;\left(\%\;P\;from\;TSP\right)=\frac{P_{cB}\;\left(mmol\right)-\;P_{0}\;\left(mmol\right)}{P_{TSP}\;\left(mmol\right)-\;P_{0}\;\left(mmol\right)}\times 100$$

(Eq. 5) $$RAE\;\left(\%\;K\;from\;K_{2}SO_{4}\right)=\frac{K_{cB}\;\left(mmol\right)-\;K_{0}\;\left(mmol\right)}{P_{K2SO4}\;\left(mmol\right)-\;K_{0}\;\left(mmol\right)}\times 100$$

### Statistics

Randomization of pots and statistical analyses were performed with RStudio, version 1.2.1355 (2019), using the package “agricolae” (Mendiburu, 2022). Data were calculated as arithmetic means ± standard error of the means of the biological replicates and visualized using the R package “ggplot2” (Wickham, 2016) and “ggpattern” (Langlais et al., 2020). Data were subjected to Levene tests for normality using the R package “heplots” (Fox et al., 2021) before a one-way analysis of variance (ANOVA) was performed. Replicate means were compared by Tukey’s honest significance test. Pearson correlations were performed using the R package “corrplot” (Wei et al., 2017). The R package “FactoMineR” (Husson et al., 2022) with the function “factoextra” (Kassambara and Mundt, 2020) was used to perform the principal component analyses and to visualize the results. The data set for principal component analyses included P extractable with H_2_O, NH_4_Cl, NaOH and HCl, as well as the mass fractions of K, Na, Ca, Mg, Fe and Al (**Supplementary Material S6**), because these elements may form phosphates in the ash and affect P extractability.

## Results and discussion

### Ash characterizations

#### Elementary compositions of bagasse-based ashes

Bagasse-based ashes were analyzed for mass fractions of N, P and K, which are the most important for plant nutrition, as well as for Ca, Mg, Na, Al and Fe, which influence the availability of P from ashes to plants. All ashes contained low mass fractions of N, which is due to N emission during thermal processing of the biomass (Mayer et al., 2022). cB and gB ashes contained similar P and K but different C mass fractions, which is due to different fuel to air ratios during thermal biomass processing. Other elements in cB and gB ashes likely differed due to soil contamination in the cB ash as it was sampled from the landfill. The chemical compositions of bagasse-based ashes were strongly modified by thermal co-processing (co-gasification and co-combustion) of bagasse pellets with the additional feedstocks of chicken manure or sewage sludge (see 2.1). In regard to P, the mass fractions increased from 0.4 wt% in ash from gasification of bagasse pellets alone (gB), to 1.7 and 2.0 wt% in gBM and cBM ashes (co-gasified and co-combusted bagasse pellets with chicken manure pellets), to 3.0 wt% in cBS ash (co-combusted bagasse pellets with sewage sludge; **Table 1**). Thus, thermal co-processing of bagasse pellets with nutrient rich chicken manure pellets or sewage sludge increased the P mass fraction in bagasse-based ashes to values also described for low grade phosphate rock (Zapata and Roy, 2004). According to European regulations (EU, 2019), gBM and cBM ashes can thus be classified as PK fertilizers since they contained more than 1.3 wt% P and 1.6 wt% K, while cBS ash could be classified as P fertilizer because of the low K content of 0.64 wt% (**Table 1**).

The extractability of P from ashes is highly affected by elemental composition and P forms (Luyckx et al., 2021). Calcium, Mg, Na, K, Fe and Al are of great importance for phosphate formation and its solubility in various extraction solutions (Kratz et al., 2019). Combining 20 wt% chicken manure pellets with 60 wt% bagasse pellets (gBM and cBM ashes), increased the mass fractions of P, K, Ca, Mg and Na in gBM and cBM ashes up to 5.0, 3.0, 9.2, 1.8 and 6.0 fold, respectively. In contrast, the mass fractions of Fe and Al decreased as much as 2.3 and 2.6 fold, compared to pure gB ash (**Table 1**). When bagasse pellets were co-combusted with sewage sludge, the mass fractions of P, Ca, Mg and Na in cBS ash increased 7.4, 4.0, 1.2 and 2.8 fold, while K mass fraction decreased 1.6 fold compared to gB ash. In contrast to chicken manure as additional feedstock, Fe was not reduced and Al even increased following the addition of sewage sludge (**Table 1**).

#### Phosphorus extractability from bagasse-based ashes

**Figure 1:**
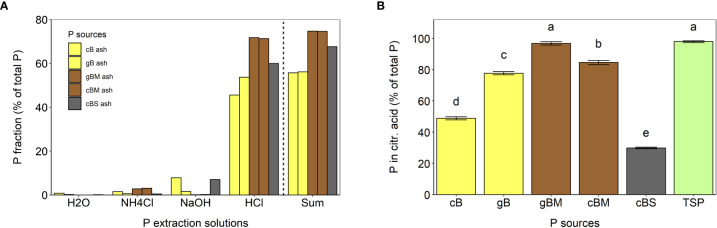
Phosphorus extractability from cB, gB, gBM, cBM and cBS ashes in various solutions. **(A)** Sequential P extractability in water, 1 M NH_4_Cl at pH 7, 0.1 M NaOH and in 0.5 M HCl. **(B)** Phosphorus extractability with 2% citric acid. The error bars represent standard errors of the means. Different letters indicate statistical differences between P extractabilities (Tukey’s HSD test, p ≤ 0.05, n = 3).

To distinguish between extractable P forms, we conducted a sequential P extraction with water, 1 M NH_4_Cl at pH 7, 0.1 M NaOH and 0.5 M HCl. The pH dependence of the extraction is thought to represent water dissolvable P, labile P, Fe-/Al-bound P and Ca-/Mg-bound P fractions (Qian et al., 2009; Brod et al., 2015). The total P content extracted by this procedure was 75% from gBM ash and cBM ash, 68% from cBS ash and 56% from cB and gB ashes (**Figure 1A**). The extractability of P from all bagasse-based ashes with 0.5 HCl was higher than all other extraction solutions (**Figure 1A**). This is consistent with previous studies reporting that P soluble in HCl is the major P fraction in various ashes (Brod et al., 2015; Lemming et al., 2020). Luyckx et al. (2021) explained this by the presence of Ca-phosphates (e.g. apatite and whitlockite), which dissolve in acidic solutions.

Only low amounts of P were soluble in 0.1 M NaOH. The highest solubility was found in cBS ash with around 7% of total P (**Figure 1A**). The different P solubility in 0.1 M NaOH was probably due to different Fe and Al mass fractions in the ashes (**Table 1**; Qian et al., 2009). The overall lowest P extractability was achieved with 1 M NH_4_Cl at pH 7 (≤ 3% of total P) and with H_2_O (<1% of total P; **Figure 1A**).

P extractability in 2% citric acid is commonly considered to predict P availability from a fertilizer for a plant (Bergfeldt et al., 2018; Herzel et al., 2020). In this study, P was extracted with 2% citric acid, yielding similar values for gBM ash (96.8%) as for TSP (98.0%), while cBM ash (85%), gB ash (78%) and cB ash (49%) contained less extractable P (% of P from total P; **Figure 1B**). This extractability of P was higher than in 0.5 M HCl, probably due to complexing agents in citric acid (Herzel et al., 2020; Luyckx et al., 2021). The lowest amount of P extracted with 2% citric acid was measured in cBS ash (30%; **Figure 1B**). Contrary to gB, gBM and cBM ashes, the extractability of P in citric acid from cBS ash was half of that seen in 0.5 M HCl, indicating the presence of different P forms in cBS ash than in other bagasse-based ashes, as well as the necessity of harsh acidic conditions for P solubilization from cBS ash.

#### Principal component and correlation analyses

**Figure 2:**
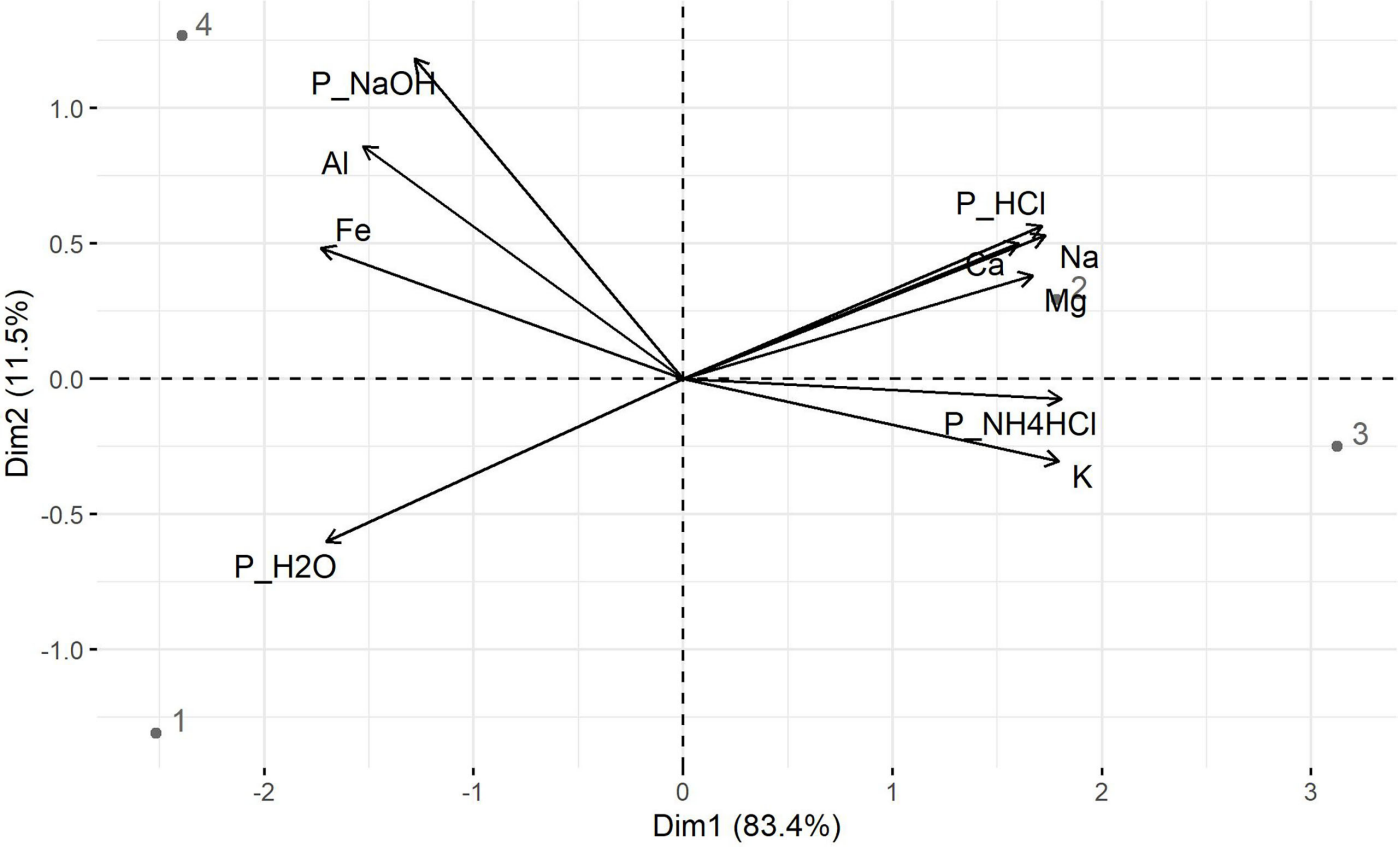
Biplot of component 1 (Dim1) and 2 (Dim2) of the Principle Component Analyses (PCA) for P soluble in water (P_H2O_), 1 M NH_4_Cl at pH 7 (P_NH4Cl_), 0.1 M NaOH (P_NaOH_), and 0.5 M HCl (P_HCl_), mass fractions of potassium (K), sodium (Na), calcium (Ca), magnesium (Mg), iron (Fe) and aluminum (Al) with gB (1), gBM (2), cBM (3) and cBS (4) ashes as sub-data set.

To understand P extractability in bagasse-based ashes, principal component analyses (PCA) were performed for P soluble in water, 1 M NH_4_Cl at pH 7, 0.1 M NaOH and 0.5 M HCl, and the mass fractions of Ca, Mg, Fe, Al, K and Na, which can form phosphates in the ash. The first two principal components, named dimension 1 (Dim1) and dimension 2 (Dim2), together explain 94.90% (Dim1: 83.4% and Dim2 11.5%) of the total variance (**Figure 2**). Interestingly, Na, K, Mg, Ca, P_HCl_ and P_NH4Cl_ formed a group with gBM and cBM ashes, while Fe, Al, P_NaOH_ and P_H2O_ formed a group with cBS and gB ashes (**Figure 2**). This indicated a close relationship between the pH of the solutions for P extraction and the mass fractions of Na, K, Mg, Ca, Fe and Al in the ashes.

Further analyses revealed that P soluble in HCl positively correlated with total Mg (R^2^ = 0.96, p ≤ 0.01), Ca (R^2^ = 0.86, p< 0.10) and Na (R^2^ = 0.82, p< 0.10) mass fractions (**Table 2A**) suggesting the presence of Mg-/Ca-phosphates and/or Mg-/Ca-Na phosphates in bagasse-based ashes. The positive correlation of K (R^2^ = 0.97, p ≤ 0.01), Na (R^2^ = 0.90, p ≤ 0.05) and Mg (R^2^ = 0.81, p ≤ 0.10) mass fractions with P soluble in 1 M NH_4_Cl hints at the presence of Mg-alkali phosphates in the ashes (**Table 2A**). Calcium- and Mg-alkali phosphates likely occurred in cBM and gBM ashes due to higher mass fractions of K and Na in the ashes compared to gB and cBS ashes (**Table 1**). High mass fractions of Fe (3.86 wt%) and Al (4.18 wt%; **Table 1**), as well as strong positive correlations with Fe (R^2^ = 0.89, p< 0.05) and Al mass fractions (R^2^ = 0.83, p< 0.1) with P soluble in 0.1 M NaOH (**Table 2A**) suggest the presence of Fe- and Al-phosphates in cBS ash.

**Table 2:** Pearson’s correlation between sequentially extracted P with distilled deionized water (P_H2O_), 1 M NH_4_Cl at pH 7 (P_NH4Cl_), 0.1 M NaOH (P_NaOH_), and 0.5 M HCl (P_HCl_) from gB, gBM, cBM and cBS ashes with total mass fractions of potassium (K), sodium (Na), calcium (Ca), magnesium (Mg), iron (Fe) and aluminum (Al) in the ashes (**A**), and with phosphorus (P) uptake from the ashes (**B**).

		(A)						(B)
Variables		K	Na	Ca	Mg	Fe	Al	P uptake
**P_H2O_**	p-value	0.42	0.39	0.22	0.12	0.04	0.34	0.05
	correlation	-0.48	-0.50	-0.67	-0.78	0.89	0.55	-0.82
**P_NH4Cl_**	p-value	<0.01	0.03	0.14	0.10	0.24	0.12	0.62
	correlation	0.97	0.90	0.76	0.81	-0.64	-0.78	0.26
**P_NaOH_**	p-value	0.15	0.40	0.35	0.14	0.04	0.08	0.18
	correlation	-0.74	-0.49	-0.53	-0.76	0.89	0.83	-0.63
**P_HCl_**	p-value	0.14	0.09	0.06	<0.01	0.03	0.21	0.03
	correlation	0.76	0.82	0.86	0.96	-0.91	-0.68	0.86

#### Crystalline P phases in bagasse-based ashes

Phosphorus phases were analyzed by X-ray diffraction (XRD) to understand P extractability from bagasse-based ashes. The crystalline P phases detected by XRD in any of the ashes contained predominantly orthophosphate and only small amounts of pyrophosphate (**Table 3**, **Supplementary Material S7**). This is in line with ^31^P nuclear magnetic resonance (NMR) analyses conducted in our study (**Supplementary Material S8**) and in a previous study by Herzel et al. (2020). The combination of ^31^P NMR analyses with sequential extraction revealed further undefined P species in the fertilizers that were not extractable with the standard extraction solution of 0.25 M NaOH and 50 mM Na_2_EDTA for ^31^P NMR analyses (**Supplementary Material S8**).

**Table 3:** Overview of crystalline P phases in gB, gBM, cBM and cBS ashes based on **Supplementary Material S7**.

P phases	gB ash	gBM ash	cBM ash	cBS ash
**AlPO_4_**				X
**Whitlockite ≈ Ca_9_M(PO_4_)_7_**	X	XX	X	XX
**CaK_2_P_2_O_7_**	X	X	X	X
**Two undefined P phases**		X	X	
**Ca(Na,K)PO_4_**			XX	

Number of “X” indicates the amount of semi-quantified P phases. The placeholder “M” in whitlockite is commonly Ca, Fe, and/or Mg (Herzel et al., 2020). Two undefined P phases were extractable in formic and/or citric acids. All crystalline P phases were soluble in 0.5 M HCl solution, while AlPO_4_ was also soluble in 0.1 M NaOH.

The P phase formation depends on the chemical elements present in the biomass before thermal processing as previously reported by Hannl et al. (2022) in sewage sludge-based ashes. In the cBS ash, high mass fractions of Fe and Al from sewage sludge (**Supplementary Material S1**) facilitated the formation of AlPO_4_, which was soluble in 0.1 M NaOH and 0.5 M HCl. High mass fractions of alkali and earth alkali metals from chicken manure favored the formation of Ca(Na,K)PO_4_ in cBM ash, and two, as yet undefined, P phases in both cBM and gBM ashes (**Table 3**, **Supplementary Material S7**). We assume that the formation of Ca-alkali phosphates in bagasse-based ashes was due to the principle of thermochemical post-treatment of ashes. In this process, ashes and alkaline additives such as Na_2_SO_4_ or K_2_SO_4_ are combusted at 1000°C for 30 min. to form Ca-alkali phosphates (Steckenmesser et al., 2017; Herzel et al., 2020). The formation of different P phases in cBM and gBP ashes may have been due to the different thermal processing environments, as illustrated by Bergfeldt et al. (2018) in ash and biochar after combustion and pyrolysis of chicken manure. All bagasse-based ashes from small-scale processing contained CaK_2_P_2_O_7_ and whitlockite (Ca_9_M(PO4)_7_), which most commonly contain Ca, K, Fe, and/or Mg at the placeholder “M”; (Herzel et al., 2020). In an earlier study, Hannl et al. (2022) detected K-whitlockite in alkali-rich ash and (Mg, Fe)-whitlockite and AlPO_4_ in Fe-and Al-rich ash. In cB ash, amorphous phases masked potential P phases (**Supplementary Material S7**).

According to earlier studies, the extractability and/or plant availability of P from AlPO_4_ and Ca-based phosphates is as follows: Ca(Na,K)PO_4_ > CaK_2_P_2_O_7_ > Ca_9_M(PO4)_7_ > AlPO_4_ (Vogel and Adam, 2011; Kratz et al., 2019; Herzel et al., 2020). Further analyses of the undefined P phases and detailed elucidation of whitlockite structure in bagasse-based ashes will help to identify the factors behind the fertilizers´ efficiency. Consequently, variation of crystalline P phases might enable prediction of plant P availability from bagasse-based ashes. Based on P extractability in 2% citric acid and XRD analyses, we predict that the fertilizer effectiveness of bagasse-based ashes for soybeans will be as follows: cBM ≥ gBM > gB > cBS > cB.

### Ash fertilization effects under greenhouse conditions

#### Effects of industrially produced bagasse ash as fertilizer

**Figure 3:**
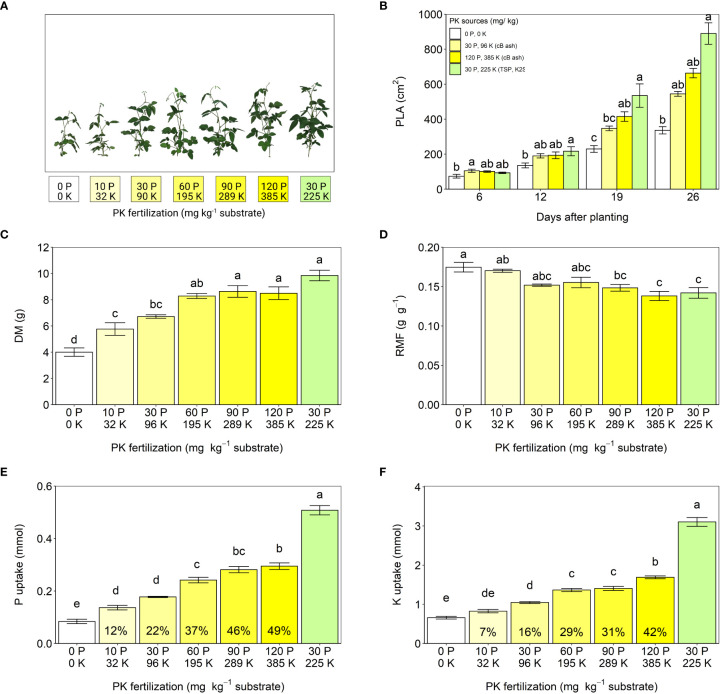
Effects of increasing cB ash doses on soybean growth (experiment 1). **(A)** Shoot phenotypes, **(B)** projected leaf areas (PLA), **(C)** total plant dry mass (DM) from shoot and root organs, **(D)** root mass fractions (RMF), **(E)** uptake of phosphorus (P) and **(F)** uptake of potassium (K) during 44 d growth. All values are given per plant. Phosphorus and potassium were provided by cB ash (yellow bars and labels) and triple-superphosphate (TSP) and K_2_SO_4_ (green bars and labels). The values within the bars **(E, F)** represent relative agronomic effectiveness compared to P and K from TSP and K_2_SO_4_. The error bars represent standard errors of the means. Identical letters indicate no statistical differences between the fertilizer treatments (Tukey’s HSD test, p ≤ 0.05, n = 5).

In experiment 1, bagasse ash (cB ash) from a sugarcane factory in Goiás, Brazil, was investigated as a fertilizer for soybeans. Compared to the non-fertilized control plants, substrate fertility was significantly increased by the addition of the ash. The measurements showing the fertilization effects included soybean shoot size, projected leaf area, dry biomass accumulation, biomass allocation to the roots, and uptakes of P and K (**Figure 3**). In general, shoot size, leaf area, biomass, and nutrient uptake increase as nutrient availability increases, while the biomass allocation to the roots decreases as plants need to forage less for nutrients in the soil (Poorter et al., 2012; Herzel et al., 2020). The positive fertilization effect of cB ash on the employed soybean is in line with earlier studies reported for various other crops, such as wheat (Gonfa et al., 2018), bean, and Chinese kale (Webber III et al., 2017).

Despite the positive fertilization effects of cB ash described above, it was less effective as fertilizer for soybeans, although 7.9 g cB ash kg^-1^ substrate provided the same dose of P as in the positive control treatment using triple-superphosphate (TSP). When compared to plants provided TSP, those receiving cB ash displayed significantly slower shoot growth 16 d after the beginning of the treatment, lower total plant dry mass, and a larger root mass fraction as well as lower P and K uptakes after harvesting the plants (**Figures 3A–F**). We assume that the fertilization effects of cB ash depended on plant P rather than K availability and N_2_ fixation, because K extractability in 2% citric acid was higher than that of P (**Table 1**), and the number of root nodules and mass fractions of N in roots did not significantly differ across treatments (**Supplementary Material S9**). The relative agronomic efficiency of P from cB ash, which presents the fertilizer effectiveness relative to referenced fertilizer (TSP in the present study), was only 22% of that of TSP (**Figure 3E**). The low efficiency is in line with P extractability in 2% citric acid (**Figure 3B**). In a study by Bogdan et al. (2021), P uptake from ashes produced by combustion of two different sewage sludges was shown to increase the relative agronomic P efficiency during seven months of ryegrass (*Lolium perenne*) growth. A long-term experiment with a perennial plant that can be harvested above ground in regular intervals, could aid in better understanding P uptake dynamics from bagasse ash and thus offer insight into the use of bagasse ash as a slow-release P fertilizer.

Increasing doses of cB ash enhanced fertilizer efficacy. Fertilization with 90 mg P/289 mg K kg^-1^ and 120 mg P/385 mg K kg^-1^ from cB ash even produced biomass yields comparable to TSP fertilization (**Figure 3C**), although P uptake again remained significantly lower than with TSP (**Figure 3E**). The use of large quantities of ash has been suggested to result in nutrient imbalances in the substrate that do not conform to crop requirements (Müller-Stöver et al., 2018). A detailed study on nutrient content in plants fertilized with increasing amounts of ash would clarify whether large cB ash doses can cause nutrient imbalances in the substrate that hinder P uptake by plants.

The pure bagasse ash tested here was not able to compete with TSP and K_2_SO_4_ for fertilizer efficiency. Future studies should investigate the effects of different local combustion conditions, times, and storage conditions on the composition, as well as the P availability from bagasse ashes to crops. This would allow better insight into how the ash could be effectively used in agriculture and industry, rather than being disposed of in landfills.

#### Effect of thermal co-processing bagasse pellets with nutrient rich residues on P-availability to soybeans

In the following, bagasse pellets were gasified (λ = 0.4) alone (gB) and in combination with chicken manure (gBM) as well as co-combusted (λ = 2.2) with chicken manure (cBM) or sewage sludge (cBS) to obtain different ashes for testing as fertilizer for soybeans under greenhouse conditions. Plant P availability from bagasse-based ashes varied and depended on the biomass used for ash production, which is in line with previous studies (Schiemenz and Eichler-Löbermann, 2010; Müller-Stöver et al., 2018).

**Figure 4:**
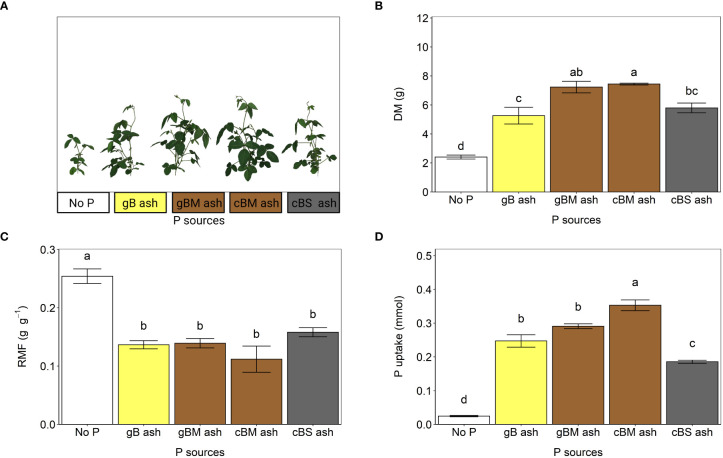
Fertilization effects of gB, gBM, cBM and cBS ashes on soybean (experiment 2). **(A)** Shoot phenotypes, **(B)** total plant dry mass (DM), **(C)** root mass fractions (RMF), and **(D)** uptakes of phosphorus (P) from the ashes. All values are given per plant. The error bars indicate standard errors of the means. Identical letters indicate no statistical differences between the fertilizer treatments (Tukey’s HSD test, p ≤ 0.05, n = 5).

The effects of pH and/or availability of other plant nutrients on plant P availability from ashes, as suggested by Schiemenz and Eichler-Löbermann (2010), were negligible in this study. The pH values in the selected substrate did not statistically differ, regardless of fertilizer treatment (**Supplementary Material S2, S4**), and N and K were not limiting due to the additional supply (see 2.3.2). Compared to gB and cBS ashes, soybeans receiving P from gBM and cBM ashes accumulated significantly more total dry biomass and took up more P (**Figures 4A–D**), indicating better plant P availability (Herzel et al., 2020). This was probably due to more Ca-/Mg-phosphates in the ashes, since P uptake is positively correlated with P soluble in 0.5 M HCl (R^2^ = 0.86, p< 0.05; **Table 2B**). Lemming et al. (2020) assumed that Ca-phosphates determine the overall level of plant P availability. Although amounts of plant dry biomass and P uptake from ashes showed evidence of different plant P availabilities, biomass allocation to the roots, which also indicates plant P availability (Poorter et al., 2012; Herzel et al., 2020), was not statistically different (**Figure 4C**). We recommend 1) further studies in arable soil to investigate plant P availability from bagasse-based ashes under field conditions and 2) the use of other crops that respond differently to ash, as shown by Schiemenz and Eichler-Löbermann (2010). Previously, Ferreira et al. (2012) reported that P extractability in Mehlich I solution from Brazilian arable soil (classified as Ferralsol) increased after fertilization with a residual ash from the co-combustion of bagasse with bovine residues.

Extraction of P from bagasse-based ashes with 2% citric acid provided a reasonable estimate for plant P availability, as indicated by plant growth analyses (**Figure 1B**, **Figure 4**). However, it tended to underestimate or overestimate P availability depending on the P phases in the ash. Phosphorus extraction in 2% citric acid predicted the lowest available P in cBS ash, which is in line with the results described above. The same extraction procedure also indicated that gBM ash should contain significantly more plant available P than cBM ash (**Figure 1**), which was not observed in the pot experiment using soybeans. Here, more P was taken up following cBM ash fertilization than with gBM ash (**Figure 4C**). The better plant P availability from cBM ash was probably due to the Ca(Na,K)PO_4_ phase in cBM ash, as it is more plant available than whitlockite (Herzel et al., 2020), which dominated in gBM ash (**Table 3**, **Supplementary Material S7**). Thus, both extraction of P in 2% citric acid and mineralogical P phase analyses are good estimates of plant P availability, although the mineralogical analyses provide a better prediction than P extractability in 2% citric acid.

The question arises whether co-processing of the low-P containing bagasse with nutrient-rich residues makes sense. Chicken manure is a valuable fertilizer in and of itself. However, the high solubility of P from chicken manure could lead to pollution of surface waters, while its combustion could reduce P solubility in water and while retaining its availability to plants (Codling, 2006; Codling, 2019). Sewage sludge ash contains high amounts of P which would only be diluted by co-processing with bagasse. In this study, these resources were used as they provided contrasting chemical compositions and were instrumental in helping to understand the P mineralogy of the bagasse-based ashes. However, careful selection of locally available residues that are produced in sufficient amounts and are otherwise potentially disposed of (as the bagasse ash itself) is indispensable for showing the benefits of using bagasse ash as a fertilizer. Finally, when such a process is implemented, life cycle analyses and economic assessments need to be conducted to investigate the environmental impacts of producing and using bagasse-based ashes as fertilizers, particularly with respect to their economic and ecological advantage over finite rock-based fertilizers.

## Conclusion

Bagasse ash represents a valuable resource that deserves attention in further studies as a potential fertilizer, with the goal of reducing the dependence on rock-based sources in fertilizer production. While the use of pure bagasse ash as a fertilizer for soybeans was limited by the low P mass fraction and plant P availability, thermal co-processing of bagasse with either chicken manure or sewage sludge increased the overall P mass fraction in the ashes to the levels of low-grade phosphate rock. The plant P availability was even shown to increase when bagasse was co-combusted with chicken manure due to the formation of plant available Ca-alkali phosphates. Thus, we recommend co-processing bagasse with alkali-rich residues to increase the P availability from bagasse-based ashes to soybeans.

## Data availability statement

The raw data supporting the conclusions of this article will be made available by the authors, without undue reservation.

## Author contributions

VD: Conceptualization, Methodology, Validation, Investigation, Writing - Original Draft. HH: Validation, Investigation, Visualization, Writing – Review & Editing. SW: Methodology, Investigation, Visualization, Writing – Review & Editing. WF-Z: Resources, Writing – Review & Editing. JWZ: Resources, Writing – Review & Editing. MM: Resources, Writing – Review & Editing, Project Administration. FM: Resources, Writing – Review & Editing. CA: Funding Acquisition, Writing – Review & Editing. HK: Writing – Review & Editing. HP: Conceptualization, Methodology, Writing – Review & Editing. NDJ: Conceptualization, Methodology, Writing – Review & Editing, Supervision, Project Administration, Funding Acquisition. SDS: Conceptualization, Methodology, Writing – Original Draft, Supervision, Project Administration. All authors contributed to the article and approved the submitted version.

## Funding

This work was supported by the German Federal Ministry of Education and Research (BMBF), within the German-Brazilian collaboration project ASHES (grant number 031A288).

## Acknowledgments

The data and scientific findings presented in this manuscript have partly been published earlier as part of the doctoral thesis by the corresponding author VD (Dombinov, 2021). The figures were compiled with BioRender.com. We are grateful for the help in plant harvest by Marlene Müller, Edelgard Schölgens and Lucy Harrison. The authors thank the team of Nova Gália sugarcane industry USINOVA in Paraúna (Goiás, Brazil) for providing information about the process, as well as for bagasse and ash samples. The authors also wish to thank Sarah Kenyon for critically reading and editing the manuscript. The authors appreciate critical reading and feedback by the referees.

## Conflict of interest

The authors declare that the research was conducted in the absence of any commercial or financial relationships that could be construed as a potential conflict of interest.

## Publisher’s note

All claims expressed in this article are solely those of the authors and do not necessarily represent those of their affiliated organizations, or those of the publisher, the editors and the reviewers. Any product that may be evaluated in this article, or claim that may be made by its manufacturer, is not guaranteed or endorsed by the publisher.

## Abbreviations

cB, combusted bagasse; cBS, combusted bagasse sewage sludge; cBM, combusted bagasse chicken manure; gB, gasified bagasse; gBM, gasified bagasse chicken manure; TSP, triple superphosphate; XRD, X-ray diffraction; NMR, nuclear magnetic resonance spectroscopy; P, elemental phosphorus; K, elemental potassium.
