# Supplementary material for: Sugarcane bagasse ash as fertilizer for soybeans: Effects of added residues on ash composition, mineralogy, phosphorus extractability and plant availability
Source: Front Plant Sci. 2022 Dec 8;13:1041924. doi: 10.3389/fpls.2022.1041924 (PMC9774024; doi:10.3389/fpls.2022.1041924)
Supplement: Supplementary file 1 [file DataSheet_1.pdf]

Supplementary Material

Sugarcane bagasse ash as fertilizer for soybeans: effects of added residues on ash composition, mineralogy, phosphorus extractability and plant availability

Vitalij Dombinov[1*], Hannes Herzel[2], Martin Meiller[3], Felix Müller[4], Sabine Willbold[5], Joachim W. Zang[6], Warde A. da Fonseca-Zang[6], Christian Adam[2], Holger Klose[1,7], Hendrik Poorter[1,8], Nicolai D. Jablonowski[1] and Silvia D. Schrey[1]

***Correspondence:**

Vitalij Dombinov

v.dombinov@fz-juelich.de

Supplementary Data

S1: Biofuels

Fuel characteristics and elementals compositions of bagasse pellets (B; Usina Nova Gália Ltda., Paraúna, Goiás, Brazil), chicken manure pellets (M; Jürgen Abeler GmbH & Co. KG, Nordwalde, Germany) and sewage sludge (S; Hohenburg, Landkreis Amberg-Sulzbach, Germany) were analyzed as follows:

Fuel characteristics: The minimum oxygen ($O_{min.}$) required to combust the biofuel was calculated according to Eq. S1, which considers the oxidation of all carbon (C) and hydrogen (H) in the biomass. The minimum air ($I_{min.}$) required to combust the biofuel was based on $O_{min.}$ (Eq. S1) and the oxygen amount in the air (Eq. S2). 0.5-1 g sample material was pressed and burned completely under oxygen supply in a calorimeter (Kalorimeter C 4000, IKA-Werke GmbH & Co. KG, Staufen, Germany) to measure Higher Heating Value (H_o). The Lower Heating Value (H_u) was calculated based on H_0 and the total amount of hydrogen, which can form water during the combustion process. The energy required to evaporate the maximum amount of water that can be formed with the available hydrogen is subtracted from the H_o. The content of water (H_2O) was determined by drying 3-6 g of sample materials at 105 °C (UFE 400, Memmert GmbH & Co. KG, Schwabach, Germany). Ash content was determined after combustion of the dried samples in a muffle furnace at 815 °C (muffle furnace L9, Nabertherm GmbH, Lilienthal, Germany). Volatiles were determined after heating 1-2 g of the sample materials in a closed pot with lid (not sealed) for 7 min. at 905 °C (muffle furnace L9, Nabertherm GmbH, Lilienthal, Germany). The volatiles could evaporate without burning the samples. To calculate the coke, water content and volatiles were subtracted from 100 % of the biomass weight. Ash subtracted from coke resulted in fixed carbon.

$$O_{min.}\,(m^3kg^{-1}) = \frac{0.01\times C\,(wt\%)}{12\,(kg\,kmol^{-1})} + \frac{0.01\,H\,(wt\%)}{4\,(kg\,kmol^{-1})} - \frac{0.01\times O\,(wt\%)}{32\,(kg\,kmol^{-1})} \times 22.43\,(m^3kmol^{-1}) \quad \text{(Eq. S1)}$$

$$I_{min.}(m^3kg^{-1}) = \frac{O_{min.}(m^3\,kg^{-1})}{0.21} \quad \text{(Eq. S2)}$$

Elementary analysis (A): 0.05-0.15 g of sample materials were combusted at 1050 °C in a CHNS elemental analyzer (Vario Macro Cube, Elementaranalysensysteme GmbH, Langenseibold, Germany) to measure carbon (C), hydrogen (H), nitrogen (N) and sulfur (S). Chlorine (Cl) was analyzed with ion chromatography (761 Compact IC, Metrohm Deutschland GmbH, Filderstadt, Germany) in the water (ultrapure water from Reinstwasseranlage arium 611 DI, Sartorius AG, Göttingen, Germany) from bomb calorimeter (Kalorimeter C 4000, IKA-Werke GmbH & Co. KG, Staufen, Germany) where chlorine was transformed to hydrogen chloride during combustion process. Oxygen (O) was the amount after subtracting water, ash, C, H, N, S and Cl amounts from 100% of the biomass weight.

Elementary analysis (B): A mixture of nitric acid (8 mL), ultrapure water (5 mL) and hydrogen peroxide (1 mL of 30 %) was added to 0.5-1 g of biomass samples (bagasse pellets, chicken manure pellets or sewage sludge) prior chemical digestion in a microwave (MLS Start 1500, Mikrowellen-Labor-Systeme GmbH, Leutkirch im Allgäu, Germany). Afterwards a mixture of hydrochloric acid (6 mL), nitric acid (2 mL) and hydrogen peroxide (0.7 mL) was added and heated in the microwave. After filtrating the digestion liquids, the elements P, S, K, Ca, Mg, Na, Mn, Zn, Fe and Al were measured with inductively coupled plasma atomic emission spectroscopy (Vista MPX, Varian, now Agilent Technologies Deutschland GmbH, Waldbronn, Germany). The properties of BM (bagasse pellets and chicken manure pellets) and BS (bagasse pellets and sewage sludge) were determined on the basis of individual fuel analyzes and brought to a ratio of 80 to 20 according to the mixing ratios.

Supplementary Table 1: Characterization of bagasse pellets (B), chicken manure pellets (M) and sewage sludge (S) alone and in combination of B with M or S at weight ratios of 80 to 20. Asterisk indicate calculated values based on measurements in bagasse pellets, chicken manure pellets and sewage sludge alone. Fuel characteristics (**A**), and elemental analyzes by CHNS elemental analyzer (**B**) and inductively coupled plasma atomic emission spectroscopy (**C**).

			**Fuels**				
	**Abbrev.**	**Units**	**B**	**M**	**S**	**BM (80:20)**	**BS (80:20)**
(**A**)							
Min, oxygen required	$O_{min,}$	$m^3\,kg^{-1}$	0.82	0.68	0.64	0.79*	0.78*
Min, air required	$I_{min,}$	$m^3\,kg^{-1}$	3.88	3.23	3.06	3.75*	3.72*
Higher heating value	H_0	$MJ\,kg^{-1}$	16.30	13.50	12.00	15.74*	15.44*
Lower heating value	H_U	$MJ\,kg^{-1}$	15.10	12.30	10.8	14.54*	14.24*
Water content	H_2O	$mg\,g^{-1}$	70.2	103.1	135.3	76.8*	81.3*
Glowing residue. ash	ash	$mg\,g^{-1}$	101.3	176.7	329.5	116.4*	140.1*
Fixed carbon	C_{fix}	$mg\,g^{-1}$	122.3	48.9	56.6	107.6*	111.1*
Volatile	V	$mg\,g^{-1}$	706.2	671.3	478.7	699.2*	667.5*
Coke (carbon + ash)	NV	$mg\,g^{-1}$	223.6	225.6	386.0	224.0*	251.2*

Abbrev.		Units	Fuels				
			B	M	S	BM (80:20)	BS (80:20)
**(B)**							
Carbon	C	mg g^{-1}	428.1	345.1	286.8	411.5*	404.1*
Hydrogen	H	mg g^{-1}	46.4	40.4	38.2	45.2*	45.0*
Oxygen	O	mg g^{-1}	349.1	275.1	152.7	334.3*	315.7*
Nitrogen	N	mg g^{-1}	2.0	52.5	48.0	12.1*	9.8*
Sulphur	S	mg g^{-1}	2.5	04.9	8.8	3.0*	3.6*
Chlorine	Cl	mg g^{-1}	0.4	2.2	0.6	0.8*	0.4*
**(C)**							
Phosphorus	P	mg g^{-1}	0.26	13.30	30.80	2.87*	5.45*
Sulphur	S	mg g^{-1}	0.26	3.87	9.14	0.98*	1.77*
Potassium	K	mg g^{-1}	1.12	18.30	2.27	4.56*	1.32*
Calcium	Ca	mg g^{-1}	1.04	50.20	36.20	10.87*	7.02*
Magnesium	Mg	mg g^{-1}	0.48	6.13	6.21	1.61*	1.45*
Sodium	Na	mg g^{-1}	0.06	1.36	0.64	0.32*	0.16*
Manganese	Mn	mg g^{-1}	0.11	0.53	0.70	0.19*	0.21*
Zinc	Zn	mg g^{-1}	0.01	0.35	1.15	0.08*	0.20*
Iron	Fe	mg g^{-1}	3.42	0.77	28.10	2.89*	7.62*
Aluminum	Al	mg g^{-1}	2.53	0.90	21.70	2.20*	5.79*

S2: Ash preparations

Bagasse ashes from the sugarcane factory ′Usina Nova Gália Ltda.′ (Paraúna, Goiás state, Brazil), hereafter named cB ash, as well as five bagasse-based ashes from small-scale combustion and gasification were used in this study. cB ash was produced by combusting 130 tons of bagasse hour^{-1} in a "horseshoe" boiler at about 1050 °C under oxidizing conditions. cB ash was washed from the boiler and transported to settling tanks. After settling and separation from the water, cB ash was stored in an open environment for air drying.

Ashes from small-scale combustion were produced from bagasse-based blends. Bagasse pellets (Usina Nova Gália Ltda., Paraúna, Goiás, Brazil) were mixed with sewage sludge (Hohenburg, Landkreis Amberg-Sulzbach, Germany) or chicken manure pellets (Jürgen Abeler GmbH & Co. KG, Nordwalde, Germany) in ratios of about 80 to 20 (w/w) without pre-treatments. Combustion of the blends was carried out in a grate furnace (HDG Compact 100 with 100 kW thermal output, HDG Bavaria GmbH, Massing, Germany) at 400 °C to 800 °C under oxidizing conditions with an air to fuel ratio (λ) of 2.2 for 45 minutes (min.). The ashes are referred to as cBS (combusted bagasse sewage sludge) and cBM (combusted bagasse chicken manure) ashes.

Based on Vodegel and Müller (2019; https://www.cutec.de/fileadmin/Cutec/documents/Thermische-Prozesstechnik/Abschlussberichte/Abschlussbericht_ASHES-CUTEC.pdf; accessed 16 August 2022), bagasse pellets (see supplementary materials A.1) were gasified in a circulating fluidized bed gasifier (ArtFuel-Gasifier, T & M Engineering, Bad Frankenhausen Germany, modified by CUTEC, Clausthal-Zellerfeld, Germany) at about 800 °C under reducing conditions (λ = 0.4) as a single fuel to produce gB (gasified bagasse) ash and as a blend with chicken manure pellets (see supplementary materials

A.1) in a weight ratio of about 80:20, resulting in gBM (gasified bagasse chicken manure) ash. Only fly ashes from the gasification process were used because they contained higher P mass fractions compared to bottom ashes, which consist mainly of bed material for which quartz sand is used (data not shown).

S3: Physicochemical effects of bagasse ash on potting media

Potting media: Chemical compositions of quartz sand (particles ≤1 mm; Rheinische Baustoffwerke GmbH, Kieswerk Inden, Inden, Germany; Nabel et al., 2018; https://doi.org/10.3389/fpls.2018.01095), a substrate consisting of quartz sand and commercial greenhouse substrate (Type: *Null-Erde*, Balster Einheitserdewerk, Fröndenberg, Germany) in a volume ratio of 1:1 (Herzel et al., 2020; https://doi.org/10.3390/agronomy10060895), and Ferralsol (46.3 wt% clay, 10.8 wt% silt, and 42.8 wt% sand) from natural and unfertilized area at Brazilian Federal University of Goiás (Universidade Federal de Goiás, UFG, Goiânia, Brazil, 16°35'27.76"S, 49°17'18.43" W) were prepared and analyzed analogously to the ash analyzes described in the manuscript (Elementary compositions). For this, *Null-Erde* was shredded (ES 300-E Roxor, FREYMATIC AG, Domat/Ems, Switzerland) and sieved (Berkili Siebmaster 1, SM1E, 18 x 8 mm sieve, HOWA parts, Warstein, Germany) before being blended with quartz sand (Herzel et al., 2020) in a volume ratio of 1:1 in a concrete mixer (ATIKA 322050, Altrad Lescha Atika GmbH, Burgau, Germany). The potting media, i.e. quartz sand, the substrate and Ferralsol soil were dried to constant weights at 60 °C (TR 1050, Nabertherm GmbH, Lilienthal, Germany) prior to analyzes.

Treatment preparations: Three potting media, i.e. quartz sand, a substrate (quartz sand and *Null-Erde*) and Ferralsol (Supplementary Table 2) in volumes of 345 mL were homogeneously mixed with five concentrations of bagasse ash, i.e. 0, 2.5, 7.5, 15, and 22.5 g L^{-1} of potting medium. The amounts of ash were equivalent to 0, 5, 15, 30 and 45 t ha^{-1} based on 20 cm tillage depth. Each fertilizer treatment contained five replicates. Substrate samples used for pH and electrical conductivity (EC) measurements were fertilized with 0 and 22.5 g L^{-1} only, i.e. 0 and 45 t ha^{-1}, and contained three replicates per fertilization treatment.

Water infiltration rate: The analyzes started with recording the dry weights of differently fertilized potting media (Supplementary Figure 1 A), and continued with water infiltration rate (WIR) measurements. Measurements of WIR were based on the single ring infiltrometer measurement reported in Bagarello and Sgroi (2004; https://doi.org/10.1016/j.still.2003.08.008) and modified as follows: Fifty milliliters of water were filled into a bottomless 50 mL Falcon tube positioned at a depth of 1 cm in the potting medium (Supplementary Figure 1 B). Measurements began after infiltration of five milliliters of water, based on the scale of the Falcon tube, and ended after infiltration of another 40 mL of water (Supplementary Figure 1 B). The infiltration rate was calculated as the infiltrated volume of water in milliliters per minute. The Falcon tube was then removed and all particles adhering to the Falcon tube were returned to the pot.

Water holding capacity: The potting media were saturated with water from the bottom of the pots for 1 h (Supplementary Figure 1 C), as described in Mangerich et al., (2015; https://doi.org/10.1021/bk-2015-1206.ch016). The weights were measured 2 h after saturation and after drying at 60 °C (TR 1050,

Nabertherm GmbH, Lilienthal, Germany) to constant weights (Supplementary Figure 1 D - G). The water holding capacity (WHC) was calculated as follows:

$$WHC\ (wt\%) = 100 \times \frac{Mass_{Wet}\ (g) - Mass_{Dry}\ (g)}{Mass_{Dry}\ (g)} \quad \text{(Eq. S3)}$$

The pH values and electrical conductivity of potting media were measured in water (1:1 *w/v*) and in 0.01 M $CaCl_2$ solution (1:2.5 *w/v*). Samples were incubated overnight at 250 rpm (TH15, Incubator Hood, Edmund Bühler GmbH, Bodelshausen, Germany) and were centrifuged at 4000 *g* for 5 min (Allegra 25R centrifuge, Bleckman Coulter, Indianapolis, United States) before measurements. The electric conductivity and pH values in water were measured with a Mettler Toledo Seven Multi (Mettler Toledo, Columbus, Ohio, USA) and the pH values in 0.01 M $CaCl_2$ with a SevenGo Duo (Mettler Toledo, Columbus, Ohio, USA).

Supplementary Table 2: Chemical compositions of the substrate (quartz sand and *Null-Erde* in a volume ratio of 1:1), quartz sand and Ferralsol soil.

**Elements**	**Abbrev.**	**Substrate ($mg\ g^{-1}$)**	**Quartz sand ($mg\ g^{-1}$)**	**Ferralsol soil ($mg\ g^{-1}$)**
**Carbon**	C	16	<0.40	12.4
**Nitrogen**	N	0.6	<0.10	1.2
**Phosphorus**	P	<0.30	<0.05	0.47
**Potassium**	K	12.72	13.4	0.63
**Sulphur**	S		<0.40	<0.02
**Magnesium**	Mg	1.04	1.02	0.221
**Calcium**	Ca	1.97	1.1	0.225
**Copper**	Cu	<0.05	<0.02	0.081
**Manganese**	Mn	0.12	0.32	0.62
**Molybdenum**	Mo	<0.03	<0.07	<0.03
**Zinc**	Zn	0.02	<0.01	0.01
**Sodium**	Na	1.87	4.16	0.07
**Silicon**	Si	380	403	170.6
**Iron**	Fe	9.38	9.5	89.8
**Aluminum**	Al	21.25	23	115.2
**Cobalt**	Co	<0.02	<0.02	0.05
**Nickel**	Ni	0.09	<0.07	0.07
**$pH_{1:2.5}$ in 0.01 M $CaCl_2$ (*w/v*)**		6.43	6.94	5.79

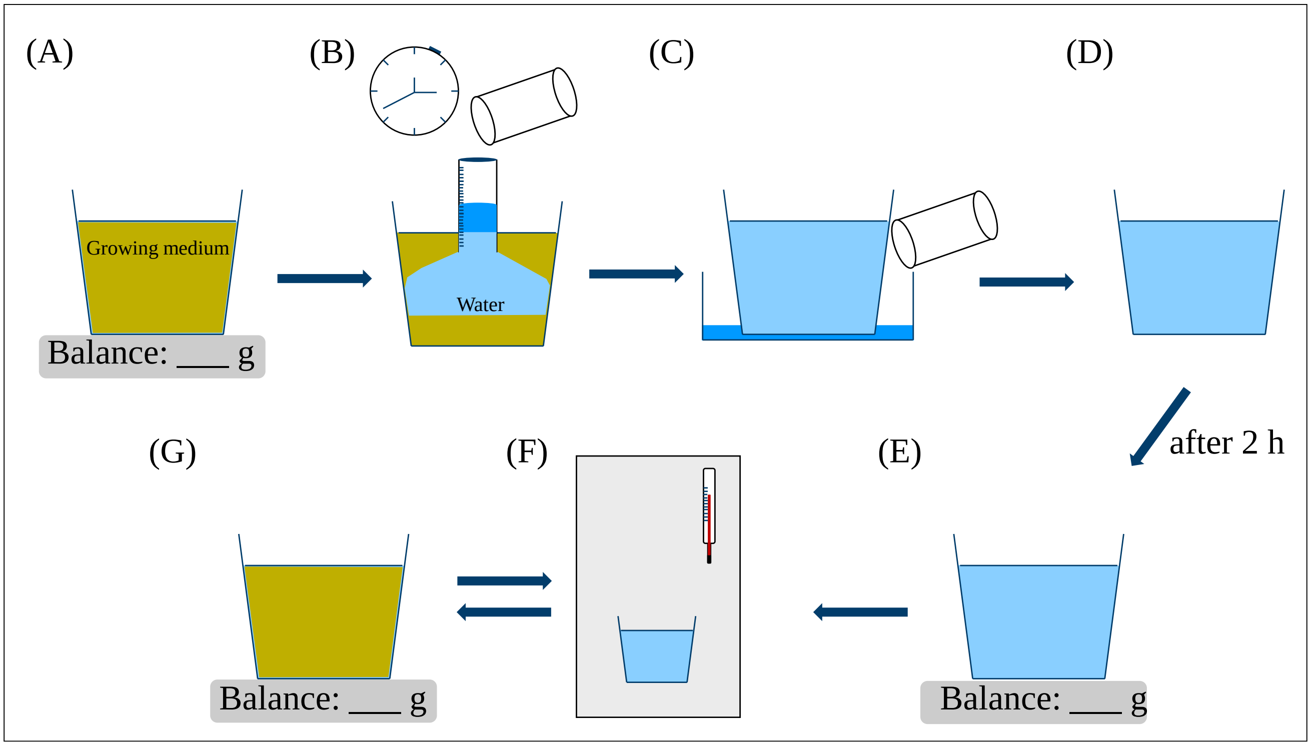

Supplementary Figure 1: Setup for measurements of water infiltration rate (WIR) and water holding capacity (WHC). (**A**) Weighing of dry potting media. (**B**) WIR: Positioning of a bottomless 50 mL Falcon tube at a depth of 1 cm. The timing started after infiltration of 5 mL water and ended after infiltrating another 40 mL. (**C**) The potting media were then saturated with water, (**D**) left for 2 h, and (**E**) measured for weight. (**F**) Drying of potting media. (**E-G**) The weights of water saturated ($Mass_{Wet}$) and dry ($Mass_{Dry}$) potting media were used to calculate the WHC.

Supplementary Table 3: Effects of bagasse ash on water holding capacity (WHC), water infiltration rate (WIR), pH measured in 0.01 M $CaCl_2$ (1:2.5 *w/v*), pH measured in water (1:1 *w/v*) and electrical conductivity (EC) in Ferralsol soil, quartz sand and the substrate (quartz sand and *Null-Erde* in a volume ratio of 1:1). All values are means ± standard error of means (n=5). Values of EC and pH in bagasse ash -free substrate and with 45 t ha^{-1} of BA contained three replicates, respectively. Identical letters indicate no statistical differences at $p \leq 0.001$ (Tukey's HSD).

Medium	BA (t h^{-1})	WHC (wt%)	WIR (mL min^{-1})	$pH_{1:2.5}$ in 0.01 M $CaCl_2$	$pH_{1:1}$ in H_2O	EC (S m^{-1})
**Ferralsol soil**	**0**	54.13^{a} ±0.72	4.56$^{n.s.}$ ±0.55	5.79^{d} ±0.04	6.04^{c} ±0.04	0.037^{c} ±0.004
	**5**	49.43^{b} ±0.02	4.34$^{n.s.}$ ±0.47	5.86^{cd} ±0.02	6.09^{c} ±0.04	0,037^{c} ±0.003
	**15**	50.24^{bc} ±0.61	4.46$^{n.s.}$ ±0.36	6.01^{c} ±0.02	6.40^{b} ±0.01	0.039^{bc} ±0.005
	**30**	49.36^{bc} ±0.50	3.31$^{n.s.}$ ±0.64	6.20^{b} ±0.04	6.53^{b} ±0.03	0.043^{ab} ±0.004
	**45**	47.75^{c} ±0.47	3.88$^{n.s.}$ ±1.45	6.50^{a} ±0.06	6.80^{a} ±0.10	0.048^{a} ±0.025
**Quartz sand**	**0**	25.64^{a} ±0.20	646.67^{a} ±43.97	6.94^{d} ±0.01	7.70^{d} ±0.03	0.011^{e} ±<0.001
	**5**	25.30^{ab} ±0.07	664.41^{a} ±22.45	7.31^{c} ±0.02	8.15^{c} ±0.15	0.014^{d} ±<0.001
	**15**	24.70^{bc} ±0.26	572.13^{a} ±14.03	7.85^{b} ±0.01	8.66^{bc} ±0.04	0.019^{c} ±<0.001
	**30**	24.07^{cd} ±0.19	425.19^{b} ±21.07	8.26^{a} ± 0.01	8.95^{ab} ±0.01	0.024^{b} ±<0.001
	**45**	23.90^{d} ±0.13	332.84^{b} ±22.38	8.31^{a} ±0.00	9.05^{a} ±0.01	0.030^{a} ±<0.001
	**0**	53.86$^{n.s.}$ ±0.51	18.80$^{n.s.}$ ±1.35	6.43$^{n.s.}$ ±0.05	6.81$^{n.s.}$ ±0.02	0.010$^{n.s.}$ ±<0.001
	**5**	55.18$^{n.s.}$ ±0.72	18.13$^{n.s.}$ ±1.10			
**Substrate**	**15**	54.42$^{n.s.}$ ±0.60	16.73$^{n.s.}$ ±1.18			
	**30**	54.50$^{n.s.}$ ±0.83	15.21$^{n.s.}$ ±0.93			
	**45**	54.11$^{n.s.}$ ±1.27	16.24$^{n.s.}$ ±0.60	6.22$^{n.s.}$ ±0.04	6.74$^{n.s.}$ ±0.05	0.010$^{n.s.}$ ±<0.001

S4: Correlation of projected and actual leaf areas

Projected leaf areas of 258 soybean plants were measured non-invasively using the ScreenHouse platform (IBG-2 Plant Sciences, Forschungszentrum Jülich GmbH, Germany) analogous to the method described in the manuscript (Non-invasive shoot growth measurements) and after harvesting the plants (destructively) using Li- 3100 (Li-cor, Nebraska, USA). Destructive measurements were conducted one day after the plant shoots were imaged by the ScreenHouse platform.

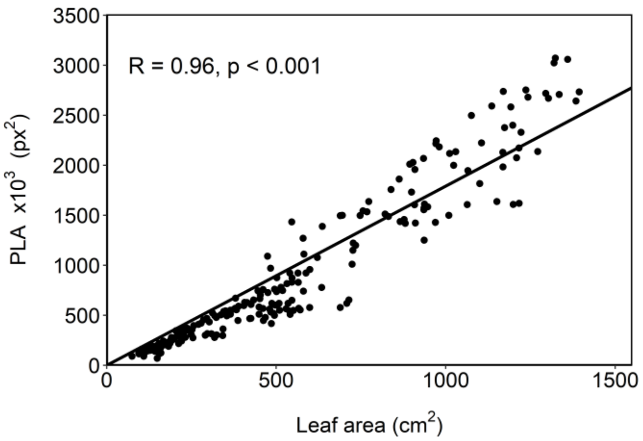

Supplementary Figure 2: Calibration curve to transform projected shoot area (PLA) from px^2 into cm^2. 1792 px^2 are equivalent to 1 cm^2.

S5: pH values in the substrates after harvesting the plants (Exp. 1 and Exp. 2)

The pH values were measured in substrates after harvesting the plants and drying to constant weight at 30°C (Destructive analyses in the manuscript). The ratio of the substrate to 0.01 M $CaCl_2$ was 1:2.5 *w/v*. It was measured according to the description in supplementary materials A.3.

Supplementary Table 4: pH values measured in the substrates (quartz sand and *Null-Erde* in a volume ratio of 1:1) after 44 d (**Experiment 1**) and 42 d (**Experiment 2**) of soybean growth. The substrate was fertilized with cB ash or triple superphosphate and K_2SO_4 (rock-based fertilizer) in experiment one and with gB, gBM, cBM and cBS ashes in experiment two. The values indicate means ±standard errors of the means (n=5).

**Exp. 1**	**0 g cB ash kg^{-1}**	**31.6 g cB ash kg^{-1}**	**TSP, K_2SO_4**
**pH**	6.21 ±0.09	6.17 ±0.02	6.07 ±0.03

**Exp. 2**	**No P**	**gB ash**	**gBM ash**	**cBM ash**	**cBS ash**
**pH**	6.20 ±0.03	6.04 ±0.06	6.24 ±0.03	6.09 ±0.05	5.84 ±0.12

S6: Principle component analyzes

Principle component analyzes were performed as described in the manuscript (Statistics). The data used for principle component analyzes is summarized in Supplementary Table 5.

Supplementary Table 5: Data set used for principle component analyzes.

Variables	Units	gB ash	gBM ash	cBM ash	cBS ash
**K**	mmol g^{-1}	0.27	0.62	0.81	0.16
**Na**	mmol g^{-1}	0.02	0.08	0.10	0.05
**Ca**	mmol g^{-1}	0.25	1.28	2.28	1.00
**Mg**	mmol g^{-1}	0.23	0.47	0.42	0.28
**Fe**	mmol g^{-1}	4.22	3.12	1.83	4.90
**Al**	mmol g^{-1}	<0.01	<0.01	<0.01	<0.01
$\mathbf{P_{H2O}}$	% of total P	0.21	0.00	0.00	0.13
$\mathbf{P_{NH4HCl}}$	% of total P	0.66	2.81	3.07	0.50
$\mathbf{P_{NaOH}}$	% of total P	1.58	0.15	0.20	7.05
$\mathbf{P_{HCl}}$	% of total P	53.70	71.78	71.37	60.02

S7: Powder X-ray diffraction analyzes

The powder X-ray diffraction (XRD) measurements were performed as described in the manuscript (Powder X-ray diffraction analysis).

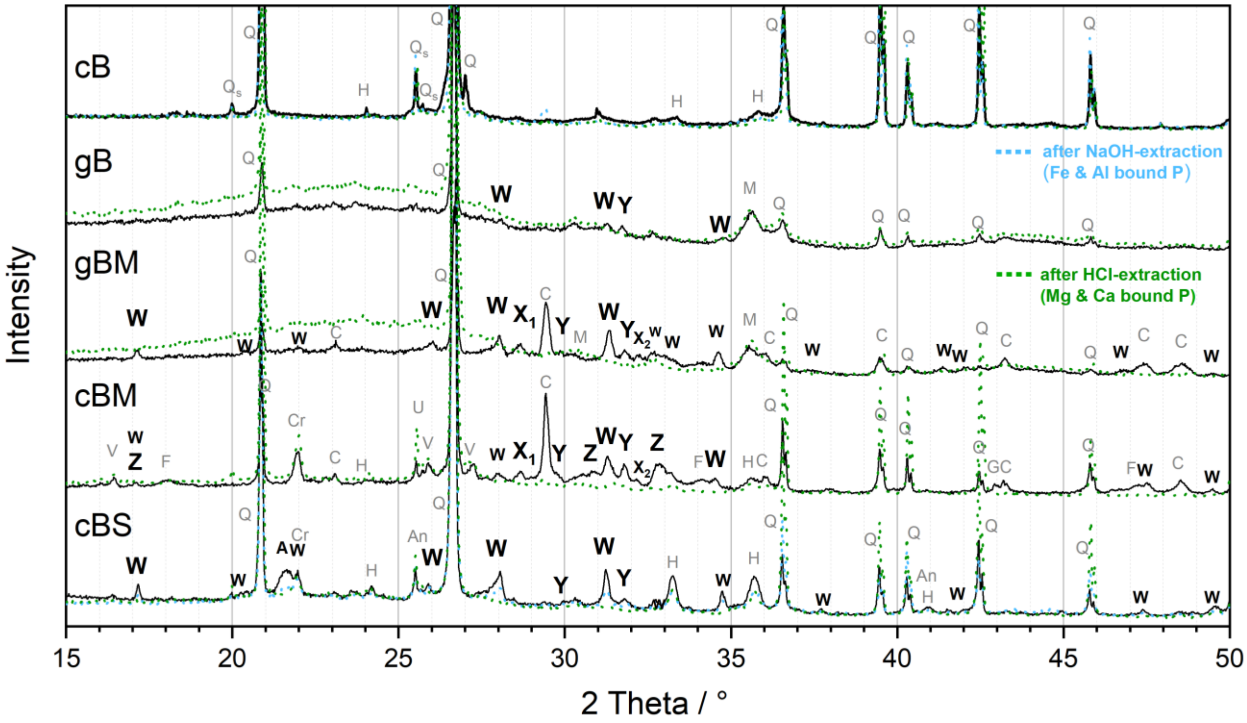

Supplementary Figure 3: Diffractions pattern for cB, gB, gBM, cBM and cBS ashes. The green dotted line is diffraction pattern of cB and cBS ashes after P extraction with 0.5 M hydrochloric acid (HCl) and blue dotted after P extraction with 0.1 M sodium hydroxide (NaOH). Phosphate phases (bold): A – $AlPO_4$, P – $CaNaPO_4$, W – Whitlockite type, X_1 – CA-/FA-soluble P-phase, X_2 – FA-soluble P-phase, Y – $CaK_2P_2O_7$ and Z – $Ca(Na,K)PO_4$. Other Phases: An – Anhydrate $CaSO_4$, C – Calcite $CaCO_3$, Cr – Cristobalite SiO_2, F – free lime $Ca(OH)_2$, G – MgO, H – Hematite Fe_2O_3, M – Magnetite Fe_3O_4, Q – Quartz SiO_2, Q_S – Quartz SiO_2 caused by stray radiation of Cu $K_ß$; W $L_{\alpha1}$/ $L_{\alpha2}$, U – $NaAlSi_2O_6$ and V – Leucite $KAlSi_2O_6$.

S8: Liquid ^{31}P NMR measurements

Liquid ^{31}P nuclear magnetic resonance spectroscopy (NMR) analyzes were performed to identify organic P species in bagasse-based ashes. Measurements were done as described in Herzel et al. (2020; https://doi.org/10.3390/agronomy10060895): P was extracted by shaking 1.5 g of dry gB, gBM and cBM ashes in 30 mL extraction solutions consisting of 0.25 M NaOH and 50 mM Na_2EDTA for 16 h. The supernatants were frozen and lyophilized. 200 mg of resulting freeze-dried solids were dissolved in a mixture of sodium deuteroxide and deuterium oxide at pH 13. Additionally, P was extracted from 5 g of gB, gBM and cBM ashes by sequential extraction (Sequential phosphorus extraction in the manuscript) to analyze P species soluble at different pH levels. The extraction solutions, i.e. water, 1 M NH_4Cl at pH 7, 0.1 M NaOH and 0.5 M HCl, were analyzed for P species without lyophilization. Methylenediphosphonic acid was added to the subsamples as an internal reference (Cade-Menun, 2005; https://doi.org/10.1016/j.talanta.2004.12.024). ^{31}P NMR spectra were obtained using a Bruker

Avance III 600-MHz spectrometer (Bruker Biospin, Rheinstetten, Germany) equipped with a Prodigy-CryoProbe.

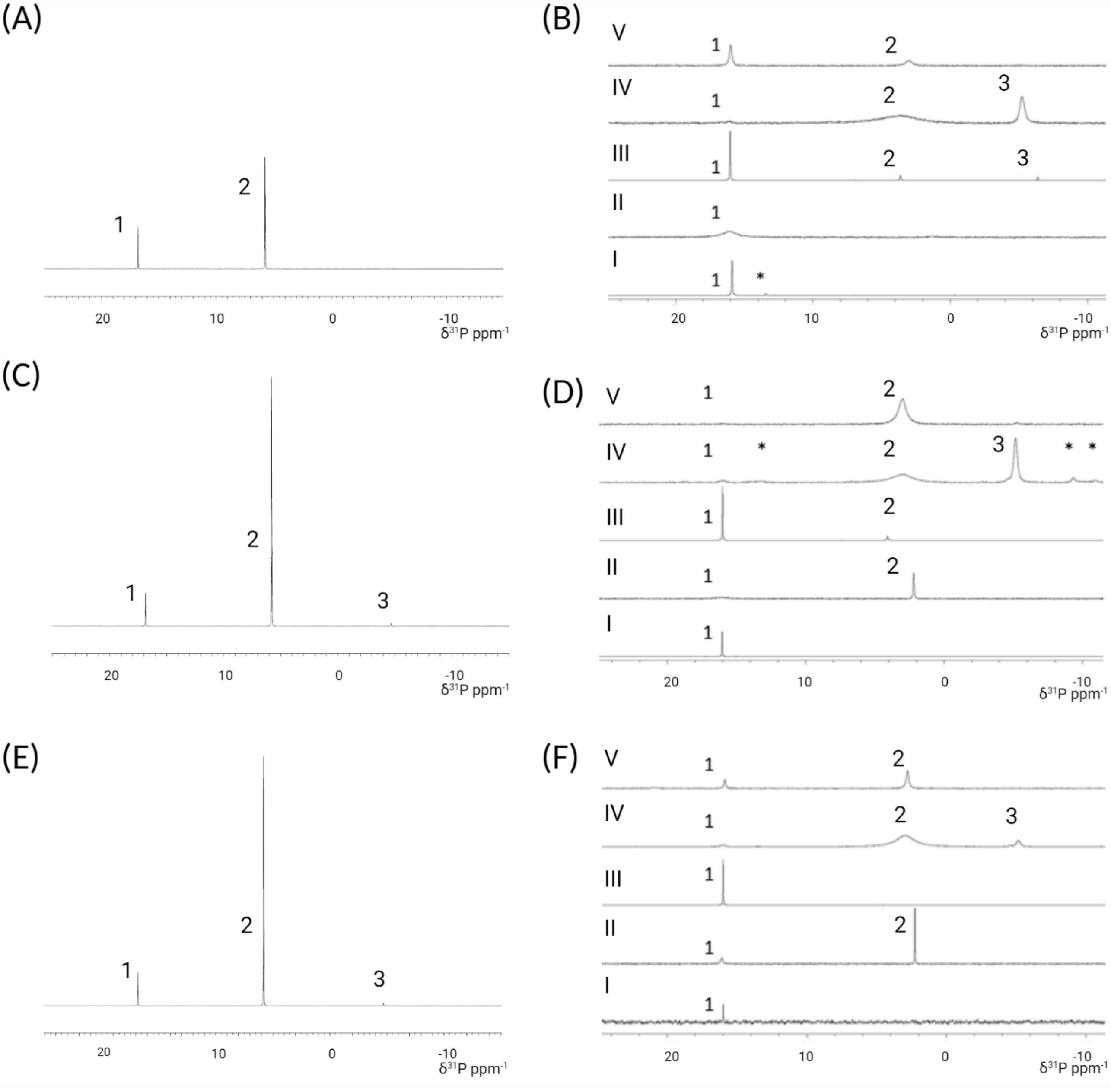

Supplementary Figure 4: Phosphorus species determined by ^{31}P NMR spectroscopy in bagasse-based ashes, i.e. (**A and B**) gB ash, (**C and D**) gBM ash, (**E and F**) cBM ash. Phosphorus extractions with 0.25 M NaOH and 50 mM Na_2EDTA: A, C and E. Sequentially extracted P with DDI water (I), 1 M NH_4Cl at pH 7 (II), 0.1 M NaOH (III) and twice with 0.5 M HCl (IV and V): B, D and F. The numbers indicate: Methylenediphosphonic acid (1), orthophosphate (2) and pyrophosphate (3). Asterisks indicate undefined P species.

S9: Nodules and nitrogen concentration in roots (Exp. 1)

In experiment 1, root nodules were counted after harvesting the plants and are presented as number per gram root dry mass. Nitrogen (N) was measured in nodulated roots in an elemental analyzer (Vario El Cube, CHN mode, Elementar Analysensysteme GmbH, Langenselbold, Germany) by combusting 2.50 mg dry biomass. Nitrogen is presented as the concentration of N in nodulated root dry mass (DM_{Root}).

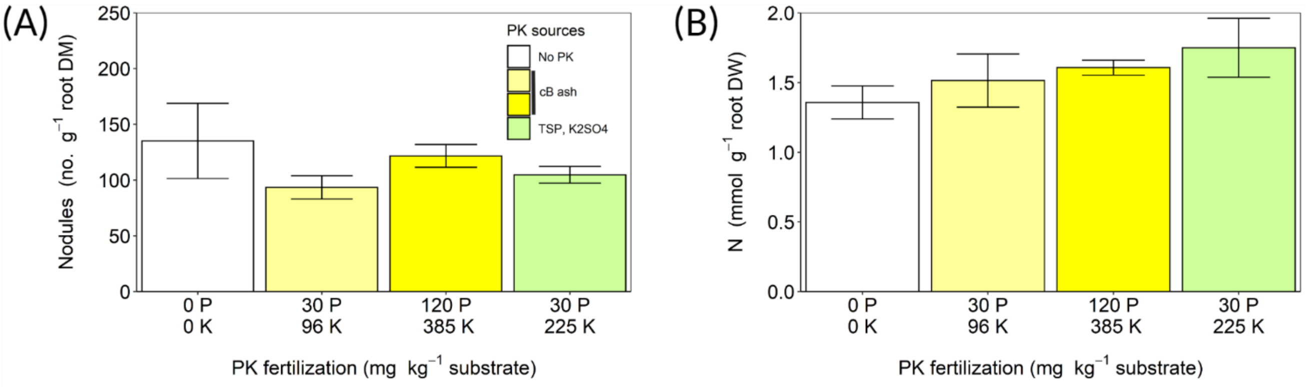

Supplementary Figure 5: Nodules numbers (**A**) and the concentrations of nitrogen (N) in roots of differently fertilized soybeans (**B**). The plants were fertilized with 0 mg phosphorus (P)/ 0 mg potassium (K) kg^{-1} substrate, 30 mg P/ 96 mg K and 120 mg P/ 385 mg K kg^{-1} substrate from cB ash, and 30 mg P/ 225 mg K from triple-superphosphate and potassium sulphate. The error bars indicate standard errors of the means. The differences were not statistically significant ($p \leq 0.65$, $n = 5$).
